# Type A fulminant *Clostridium perfringens* sepsis indicated RBC/Hb discrepancy; a case report

**DOI:** 10.1186/s12879-019-4350-3

**Published:** 2019-08-15

**Authors:** Masahide Sakaue, Koshi Ota, Eriko Nakamura, Masahiko Nitta, Masahiro Oka, Yasuo Oishi, Yohei Sano, Shinya Yonogi, Akira Takasu

**Affiliations:** 1 Department of Emergency Medicine, Osaka Medical College, 2-7 Daigaku-machi, Takatsuki City, Osaka 596-8686 Japan; 2 Osaka Institute of Public Health, Osaka, Japan

**Keywords:** *Clostridium perfringens*, Hemolysis, Liver abscess, Shock, Sepsis

## Abstract

**Background:**

*Clostridium perfringens* can cause various infections, including food poisoning, gas gangrene, cellulitis and fasciitis. *C. perfringens* septicemia is rare, but is a known cause of hemolysis by damaging red blood cell, and often proves rapidly fatal in emergency department (ED) situations.

**Case presentation:**

A previously healthy 76-year-old man presented to the ED 8 h after onset of acute abdominal pain and diarrhea. Laboratory examination revealed a large discrepancy between the red blood cell count of 1.91 × 10^6^/mm^3^ and the hemoglobin level of 10.3 g/dL, suggesting massive intravascular hemolysis. Computed tomography revealed liver abscesses with gas. During ED treatment, the state of the patient rapidly deteriorated and he entered cardiopulmonary arrest. Blood cultures finally identified *C. perfringens*.

**Conclusion:**

Intravascular hemolysis and red blood cell (RBC) / hemoglobin (Hb) discrepancy in the presence of infection should prompt ED physicians to consider *C. perfringens* septicemia and to act quickly to provide appropriate treatment.

## Background

*Clostridium perfringens* is one of the most common causes of food poisoning, but can sometimes be curable if treated within a few days. *C. perfringens* septicemia is a rare, but rapidly fatal disease with a reported mortality rate of at least 60% [1].

*C. perfringens* type A produces an α-toxin that induces hemolysis by destroying red blood cells, resulting in a failure to supply oxygen to tissues [2, 3]. Hemolysis with signs of septic shock due to *C. perfringens* infection has been reported as invariably fatal in several reports [1, 4, 5]. We report herein a case of fulminant *C. perfringens* septicemia with massive acute hemolysis and liver abscess. Despite rapid initiation of antibiotic therapy, the patient died within 3 h of admission to the emergency department (ED).

## Case presentation

A previously healthy 76-year-old man presented to the ED 8 h after onset of acute abdominal pain and diarrhea. He had eaten no perishable foods, and no family members had shown any symptoms. His medical history only included endoscopic submucosal dissection for early gastric cancer 6 years prior to the presentation. No family history of note was elicited.

On arrival, the patient looked unwell and flushed. Vital signs were: temperature, 38.6 °C; heart rate, 117 beats/min; blood pressure, 176/93 mmHg; and respiratory rate, 26 breaths/min. Peripheral oxygen saturation was 95% on room air. Abdominal examination revealed mild tenderness in the periumbilical region. The neck was supple. Heart sounds were normal, the lungs were clear to auscultation, and results of neurological and skin examinations were unremarkable. Arterial blood gas analysis revealed metabolic acidosis and compensatory respiratory alkalosis (pH, 7.486; partial pressure of arterial carbon dioxide, 16.1 mmHg; partial pressure of arterial oxygen, 66.1 mmHg; lactate, 86.4 mg/dL; base excess, − 9.2 mmol/L). The blood sample showed hemolysis, which was initially ascribed to an error during collection. Laboratory examination revealed a discrepancy between the red blood cell count (RBC) of 1.91 × 10^6^/mm^3^ and the hemoglobin (Hb) level of 10.3 g/dL. Other data were as follows: white blood cell count, 20,900/mm^3^ with 85.6% neutrophils; C-reactive protein, 4.70 mg/dL; procalcitonin, 28.82 ng/mL; platelet count, 545,000/μL; blood urea nitrogen, 27 mg/dL; creatinine, 2.42 mg/dL; serum aspartate aminotransferase, 874 IU/ L; serum alanine aminotransferase, 299 IU/L; and lactate dehydrogenase, 7605 IU/L; Na, 124 mEq/L; Cl, 92 mEq/L; K, 5.8 mEq/L.

**Fig. 1:**
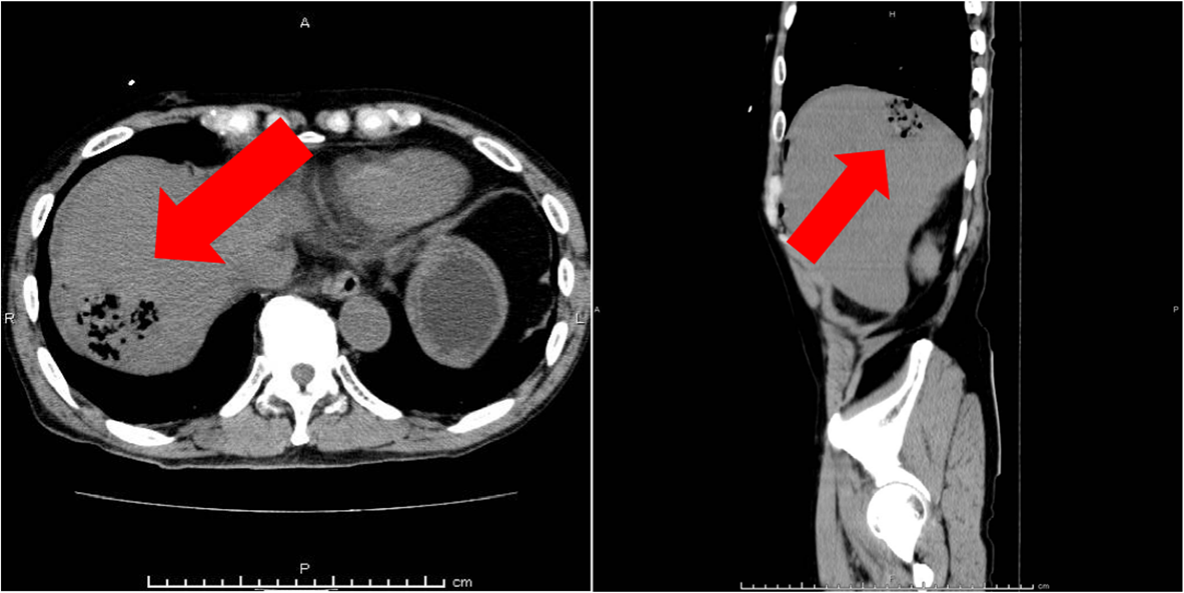
Computed tomography shows abscesses with gas in the right posterior lobes of the liver (red arrow)

Computed tomography performed 45 min after arrival in the ED revealed abscesses with gas in the right posterior lobes of the liver (Fig. 1). In light of this imaging result, he was treated empirically for suspected septicemia from liver abscesses with 1 g of meropenem after blood cultures were obtained.

The decision for immediate admission to the intensive care unit was made after consultation with the general surgery service to plan drainage of the abscesses. He was treated with fluid replacement of 1000 mL. Despite these ED workups, however, his condition rapidly deteriorated and cardiopulmonary arrest occurred suddenly, 150 min after arrival in the ED. Attempts at cardiopulmonary resuscitation proved unsuccessful and the patient died. Two sets of 10-mL blood had been obtained (1 each for the aerobic and anaerobic bottles) on his arrival and submitted to our central laboratory after standing at room temperature for 12 h. The bottles were incubated for 48 h using the representative automated blood culture system (BACT/ALERT 3D; bioMérieux, Inc., Durham, NC) and *C. perfringens* was identified by the anaerobic blood cultures.

Multiplex polymerase chain reaction only identified expression of the α toxin gene, and the pathogen isolated from the present patient was shown to be *C. perfringens* type A (Table 1).

**Table 1:** Multiple PCR showing only α toxin gene

sample	α-toxin	β-toxin	ε-toxin	ι-toxin	CPE	NetB		BEC
1	+	–	–	–	–	–		–

*CPE C. perfringens* enterotoxin, *NetB* necrotic enteritis B-like toxin, *BEC* binary enterotoxin of *C. perfringens*

## Discussion and conclusions

*C. perfringens* septicemia is rare and typically proves rapidly fatal, with a reported mortality rate of at least 60% [1]. This expeditious lethality is due to a combination of the 7-min doubling time of the organism and the production of a multitude of virulent toxins. *C. perfringens* is classified into 5 types (types A–E) based on the production of different major lethal toxins [6]. All types of *C. perfringens* produce α toxin [3], which has been reported to hydrolyze phospholipids in red blood cell membranes, leading to massive hemolysis and severe tissue hypoxia due to the failure of oxygen transport functions [2, 5]. This may cause RBC/Hb discrepancy. The complete blood cell count machine can measure hemoglobin despite the hemolysis, however, it cannot measure the lysed red blood cells.

*C. perfringens* is an anaerobic Gram-positive bacillus usually found in the human gastrointestinal tract, and is most well-known as the cause of gas gangrene [1]. Several reports have described fatal *C. perfringens* septicemia associated with liver abscess [1, 7, 8]. Although the exact mechanisms of entry into the blood stream remain unknown, bacterial translocation may occur via portal veins in proximity to abscesses in the liver [9].

The key treatments for this rapidly lethal infection include immediate administration of antibiotics and surgical intervention. In the present case, the patient deteriorated rapidly and died despite administration of a broad-spectrum antibiotic soon after arrival. The ED clinical course was catastrophic and he was not a candidate for surgical intervention. Nishikido and Hashiba have also reported rapidly fatal ED cases despite early antibiotic administrations [1, 5].

Intravascular hemolysis and RBC/Hb discrepancy in the presence of infection should prompt ED physicians to consider *C. perfringens* septicemia and to act quickly to treat the infection.

## Data Availability

N/A

## Abbreviations

BEC: Binary enterotoxin of *C. perfringens*
CPE: *C. perfringens* enterotoxin
ED: Emergency department
Hb: Hemoglobin
NetB: Necrotic enteritis B-like toxin
RBC: Red blood cell

## References

[1] Nishikido T, Tamemoto H, Kurasawa M, Koike J, Tominaga S (2017). Gas-forming liver abscess associated with rapid hemolysis in a diabetic patient. World J Diabetes.

[2] Ohtani K, Shimizu T (2016). Regulation of toxin production in Clostridium perfringens. Toxins (Basel).

[3] Rood JI, Adams V, Lacey J (2018). Expansion of the Clostridium perfringens toxin-based typing scheme. Anaerobe..

[4] van Bunderen CC, Bomers MK, Wesdorp E, Peerbooms P, Veenstra J (2010). Clostridium perfringens septicaemia with massive intravascular haemolysis: a case report and review of the literature. Neth J Med.

[5] Hashiba M, Tomino A, Takenaka N (2016). Clostridium perfringens infection in a febrile patient with severe hemolytic anemia. Am J Case Rep.

[6] Goadsby PJ, Kurth T, Pressman A (2016). HHS Public Access.

[7] Paasch C, Wilczek S, Strik MW (2017). Liver abscess and sepsis caused by Clostridium perfringens and Klebsiella oxytoca. Int J Surg Case Rep.

[8] Kishi T, Miura G, Tsukuda T (2011). A case of liver abscess caused by Clostridium perfringens. Japanese J Clin Radiol.

[9] Cochrane J, Bland L, Noble M (2015). Intravascular hemolysis and septicemia due to Clostridium perfringens emphysematous cholecystitis and hepatic abscesses. Case Rep Med.

